# Water: Its History, Characteristics, Hygienic, and Therapeutic Uses

**Published:** 1861-11

**Authors:** Samuel W. Francis

**Affiliations:** New York


WATER: ITS HISTORY, CHARACTERISTICS, HYGIENIC, AND THERAPEUTIC USES.—By *Samuel W. Francis*, A. M., M. D., of New York.—Steam, the brother of water, lived in the bowels of the earth for centuries ere man discovered its utility and mastered its power. So great an agent; so hidden a virtue; so unaccountable a force required the lapse of nineteen hundred years before the extent of its conduciveness in relieving labor was fully appreciated.

What a vast field of discovery and experiment exists between the simple Eolipyle of Hero,* over a century before the Christian era, and the latest inventions of Watt, Fulton, and Stevenson!

Savory first successfully substituted steam for the labor of animals. Newcomen first applied it to a more solid body. Watt made the first steam engine. Fulton applied it first to propelling vessels in water. Evans and Trevithick first adapted it for locomotive on land."†

What mighty intellects; what determined perseverance; what sacrifice of time; what loss of life; what waste of powerful energies; what superhuman efforts did steam exact as equivalents for submitting to be confined within the narrow limits of a business life. From its birth accustomed to mount up to the purest ether and dwell amid the regions of a softer clime, it could not yield to sordid views and work for what would never reach to heaven. The cause of its subjection, found only in the gloomy abodes of earthy dwellings—gold—was not sufficient recompense for the gentle, cloud-like form of its mist-like beauty, free as air and pure as nature's own adopted child. And even now, unlike wild animals that can be conquered and completely broken in, never more resisting, steam seeks every opportuuity to escape; and, while working with an hundred horse power, strives to burst her bonds, reach a purer atmosphere and leave all sublunary objects to a feebler power. The discovery of hidden wealth has brought renown upon the finder and benefited much the country that possessed the mine; the successful wars of mighty leaders have proclaimed their names as great—time-honored heroes that they are; the appearance of a new continent in 1492 A. D., rendered immortal the name of Christopher Columbus; but the discovery and application of steam—the soul of water—has

---

* Historical and Descriptive Anecdotes of Steam Engines, etc., by Robt. Stuart, London, 1829.

† Renwick on the Steam Engine.

heaped blessings on mankind, and taught us to revere the element water in all its threefold beauty and importance.

In *Therapeutics* water must of necessity stand more or less preëminent. In the first place, no remedy, be it ever so mild, can be swallowed without the use of a liquid to assist the muscles of deglutition in contracting on the fluid and thus forcing it down. Where a solid substance is administered, the act of swallowing can not be performed without the aid of the salivary glands, with their apportioned duties, of no small importance to the patient or the man of healthy parts.

In viewing the phenomena of disease in all its different forms, and diagnosticated in every type, be it malignant or of a simple character, we find that the treatment which the medical man is enabled to prescribe is to be found principally in the Pharmacopiæ, and on reading closely the list of remedies, and comparing the different methods adopted by the learned of various countries, one can not help being struck with this all-important fact, namely; The desire of "washing away the disease" in one way or another, as the case may demand, or the symptoms indicate. Thus we have diuretics to force the kidneys to secrete more rapidly the urine, and carry off much of the noxious materials of the blood, whether it be the urea, or the dead plasma, which, being used in the destruction of blood discs, occasioned by musculary exertion, is now to be eliminated by the malpighian tufts. And here we find that on analyzing urine, out of one thousand parts, eight hundred are water, and the remaining one-fifth is made of urea, uric acid, sulphates, phosphates, and chlorides, in solution.

But should the kidneys be affected by disease, should acute nephritis set in, or the sufferer be afflicted with Bright's disease in any form, and the practitioner be consulted for his medical advice; as we know there exists a marked sympathy between the urinary organs and the lungs, and more particularly between them and the skin, its action in summer taking, to a great degree, the labor from the kidneys, and persons, consequently, micturating less frequently in the hot months than in winter, when the little capillaries are closely contracted by cold, the remedy at once recommended, suggesting the proper means for allowing the kidneys to rest in order to recover their normal power, is a diaphoretic. By this means, and in any congested state they tend, in inflammatory diseases to allay irritation, produce a sudorific effect, and literally sweat off the disease thereby, in connection with the proper anti-phlogistic measures of relieving the patient from the great congestion necessary to inflammation, as in pneumonitis, bronchitis, pleuritis, and other diseases of the thoracic organs.

Where there is active congestion, in the first stages of inflammation, resulting from external violence, the application of cold water dressings causes the capillaries to contract powerfully, forces back the rush of blood to the injured part and the sufferer recovers rapidly. But where these little tubules have been stretched until their contractile power is lost, and become, as it were, asthenic, then bathing the wound or bruise with warm water allays pain by its more soothing influence, and opens the pores of the adjacent part, promoting exudation where the blood vessels were in a state of passive congestion. Schmucker (1712–1786) based much of his treatment of wounds of the head upon the judicious application of cold water. Likewise Lombard, (1741–1811,) together with Percy, (1754–1825) derived a great deal of useful information from a miller, in the Elsass, 1785, who assisted the surgeons in dressing the wounds of the soldiers, in Strassburg, with a solution of alum and water, which circumstances led Percy subsequently to assert that if Sydenham "would not be a physician without opium, he should not like to be a surgeon without water."

Cold water dressings in surgery are esteemed of great practical utility; and, in many cases, become indispensable to the surgeon while in attendance upon the suffering. Though Hippocrates and Celsus were most energetic in behalf of the use of cold water, the sentiment appears to have escaped notice and practical use till Ambrosius Paré (1509–1590,) discovered anew its tonic properties, while searching for the Italian balsamic oil, which, at one time, wrought such marvelous cures in the healing of wounds and sluggish sores. Soon after this Jaubert and Martel (1600) maintained its wonderful power in alleviating pain. In France, Chirac and Lamorier (1730) proclaimed its virtues; and, in Italy, Sancassani, (1659–1737,) Benevoli, (1685–1756,) and Caldini, (1724–1813) announced their belief in the stimulating and medicinal properties of certain qualities and quantities of water.

Freezing an extremity with ice and salt at once deadens sensation by benumbing the nerves, rendered by irritation and consequent tendency of blood to the part, which distends the vessels, and thereby presses upon their thin, attenuated fibres, much more susceptible to pain; and the removal of a splinter from beneath the nail may be effected without the knowledge of the patient. Water-beds, in cases of fracture, or where paralysis has destroyed the power of motion, render the sufferer more comfortable at the time, and prevent, to a great degree, those most incurable of all troubles affecting the sick and enfeebled, namely, bed-sores, which, in too many cases, proclaim the rapid decline of the patient toward the grave. As slight as it may appear, and as common to the person of cleanly habits, frequent ablutions of the body remove the susceptibility to disease, and promote a greater amount of superficial circulation, which renders the man of health better able to cast off any offensive gas that may either have been generated in the system or received by inspiration into the lungs.

In Dr. Leigh's History of Lancashire, mention is made of some chalybeate waters, which he asserts to have found more efficacious in the treatment of "leprous distempers, scorbutic atrophy, the rickets, and scorbutic rheumatism," than any known medicine in his day. And Dr. Floyer remarks, in his "Fourth Letter," etc., "If cold baths are proper for the scurvy and consumption, then they are useful in the several species and complications of them with other diseases."

Were persons to preserve a scrupulous cleanliness of habit, and follow more faithfully than is their wont, the simple laws of common-sense hygiene, doctors' fees would be materially lessened, and that inability on the part of the sufferer to throw off the disease would no longer be an additional cause of prostration, which not unfrequently terminates in typhus.

When it is remembered that twenty-eight miles and upward of pores carry off much of the impurities of the blood to the surface of the body, where, if not properly removed by a free use of water, they must remain deposited, and close up their little outlets, it may readily be seen how indispensable it is for man to remove, by frequent cleansing, all that may serve to arrest the progress of this effort of nature to free itself of any morbific tendencies.

We are indebted to Lavoisier and Seguin for some valuable data relating to the quantity of vapor excreted from the surface of the body and exhaled from the lungs. After many careful and elaborate experiments, Seguin especially ascertained that the maximum amount of pulmonary and cutaneous exhalation, in twenty four hours, amounted to five pounds; the minimum, owing to one or more special causes, one pound, eleven ounces, and four drachms, (xlii. 1790.) Valentin, by exact measurement, discovered that a person, in a normal state of health, who consumed each day forty thousand grains of food and drink, exhaled from his skin and lungs some three and a half pounds. By making due allowance for the weight of the carbonic acid, and deducting all that may come from the respiration, two and a half pounds are found to be the diurnal exhalation of the skin. Much of this transudes, if we adopt Krause's " estimate of about eight square inches for the total evaporating surface of the sudoriforous glands." Hence we can not fail to see the urgent necessity of removing, as soon as may be, the accumulation of any foreign material that, by its presence, prevents a free transpiration, etc. in future.

Most of the diseases the physician is called upon to treat, in a slightly modified form, begin with inflammation in the first stage of congestion and the consequent symptoms that ensue. As the disease advances, either lymph or serum is cast out through the distended vessels ; and, lastly, absorption or some temporary effort of the organ affected, to free itself from the foreign matter, ends the case.

As for example, let the pleura be taken as a type of inflammatory action. The first cause of pain is that occasioned by the dryness of the two surfaces in the congested state. The friction sound that the auscultator hears so distinctly is caused by the movement of the sides of the pleura at each inspiration and expiration. And now, in pleuritis, the normal amount of fluid (composed chiefly of water!) that so beautifully lubricates the surfaces, has disappeared, and this unusual attrition of parts brings about that peculiar shooting sensation in respiration which is so distressing to the patient, and at once demands speedy relief. In the second stage, the serum of the blood is effused into the sac formed by the two membranes, and thus compressing the lung of the affected side throws double duty upon the healthy lung, and not unfrequently brings on, by this additional labor, emphyzema—at times producing death by apnœa, or by displacing the heart, and, consequently, accelerating the pulse, syncope terminates the life of the already exhausted patient. The serum contains, as its principal ingredient, water, and, if not removed by absorption, increased by the judicious administerings of antimony, Dover's powder, nitrate of potash, acetate of potash, or any of the diuretics or diaphoretics, this serum, with more or less lymph, which may have exuded through the little capillaries, will degenerate into pus; and empyœmia may call for paracentesis thoracis to lessen the complication and save life, which is rapidly declining under hectic symptoms of an aggravated character. It is curious to the thoughtful that this disease, rendered dangerous by the excess of fluid effused into the pleural sac, is also caused, in a great measure, by the prompt use of such remedies as shall promote a free diaphoresis and continued secretion of the kidneys, and likewise by the moderate use of *hydra-gogue* cathartics.

Thus also with the brain, encephalitis presents an unfavorable prognosis from the fear of effusion, which may terminate in hydrocephalus, and, by the constant pressure upon the brain, produce death in a more or less speedy manner.

Diseases of the heart, apart from auscultation, are recognized by the appearance of anasarca; and so invariably are the two associated that an able practitioner of this city said, that "tight boots at night" may be considered as one of the certain proofs of cardiac trouble. And not unfrequently is the patient "drowned in his own fluids" ere the valves of the heart have ceased to perform their functions, disordered by atheromatous deposits of hypertrophy, occasioning dilatation and consequent regurgitation. Chronic disease of the kidneys may be diagnosed, together with other symptoms, by the presence of dropsy, œdema of the inferior papebral sinus, being an additional proof of the existence of renal disorder.

And when that largest of all organs in man, so much abused by him in his excesses of enjoyment and epicurian mode of living, is diseased, one of the strongest signs of cirrhosis, after palpation has revealed the decrease of size, and nobby feel of its surface, is ascites, resulting from the chronic congestion of the liver; consequent engorgement of the portal vein, and likewise tension of the mesenteric veins. The capillaries can not resist the increased amount of pressure from within, and serum is effused—the liquor sanguinis being the watery composition in blood.

Curious moral is it indeed, when man seeks to live beyond his capacity, and overtaxes what has been provided for moderate enjoyment, neglecting the much revered precept, "*Maden agan*" of Aristotle, to gratify a pampered and depraved appetite; when he passes hours at the sumptuous repast, and partakes too freely of alcoholic stimulants, till the brain reels from the excess of carbon in the blood; his last desire before consciousness vanishes is to satisfy a parching thirst; and now he pushes aside the rich o'erflowing goblet, crimson, a proper type of another victim slain for Bacchus, and seeks the cool, unequaled water, to refresh his burning throat and inflamed imagination. This is but the result of intemperance so well described below:

* "Of all the evils darkening here below,
Thy hand, intemperance, works the direst wo!
Could all the gathered tears attest thy might,
Oh what a sea would welter on the sight!
Could all the moans be heard from thy career,
What a wild sound would peal upon the ear!
Could all thy victims march in dread array,
Across the world would stretch their blackening way.
Thine, the poor drunkard, reveling in his shame;
Thine, the young bride that bears his blighted name."

Such are the poet's sentiments! He lives a drunkard and dies of dropsy. The result a hob-nailed liver. In metaphor each nail resulting from a separate debauch, and significantly typical of his driving with each successive drink a nail into his future coffin lid.

In apoplexy, where the effusion of blood into the encephalon renders the life of the patient of doubtful issue, the application of bags or bladders of ice to the head, lessens the dull, heavy pressure, and serves to drive out the blood through the smaller vessels from the center of thought. Acute gastritis is oftener allayed by swallowing small pieces of ice, than by administering the multifarious decoctions and much abused opiates, which only deaden consciousness and relax the system, but can not reach the inflamed stomach as readily. This, the latest treatment, applied to cases of a similar character, dates as far back as the time of Themison, a disciple of Asclepiades (50 a, chr.) who was accustomed to reduce all treatment to the principle of contraction and relaxation, and whose follower and admirer, Eudemus, strongly advised cold clysters as a remedy for the alleviation of gastralgia.

Celsus (23 p. chr.) advocates the use of water as a bath, affusion, or even in the capacity of a beverage, for almost every disease of the alimentary canal, and, what is most cu-

* Alfred B. Street.

rious, even when the patient is afflicted with that most agonizing of diseases, hydrophobia.

The strong prejudice against the use of cold drinks, and especially that of cold water in fevers, or when the usual dose of mercury has been given, is greatly removed by the more enlightened and a wider experience in philosophical investigations and practical truths, deduced from facts of a most interesting character.

It is found pleasing to the taste, cooling in its effects, and productive of a freer diaphoresis. And the moderate sponging of the body after a long and protracted illness, with warm water and soap, promotes a more healthful and certain circulation, which takes off, as it were, an oppressive load from the system. This was urged by Rhazes (923), whose boldness of conception was only equaled by his originality of thought. In phthisical persons, where tubercles have filled up portions of the lungs, and percussion reveals a marked dullness near the apices, a free use of the sponge bath greatly assists the breathing, which has for its immediate object the æration of the blood; and if any of the onus can be removed by the skin, acting in connection with the pulmonary apparatus, much benefit is derived, and life is positively prolonged.

Even the Emperor Augustus, following the advice of the learned Antonius Musa, abandoned the enfeebling "fur-lined apartments," which he had occupied from a fear of approaching phthisis, and, by a judicious use of water, braced his system, and recovered tone. To this same Musa was the susceptible Horace indebted for much refreshment after many a night's "three times three," by the stimulating influence of a cool bath. I would not have it thought that I entertain any of the views of those over-enthusiastic so called "hydropathists." Such is not the case. It is only my object to trace out the uses, history, and interesting details connected with the characteristics of water, that lead me to enter into the opinions of the past and present practitioners, as regards the purity and indispensible qualities of all that may pertain to it.

Among the many diseases to which man is heir may be enumerated hydrocele, hydatids, hydro-thorax, hydro-cephalus, hydro-pneumo-thorax, and many others that terminate in dropsical effusion. Hydrophobia is one of a special character, no affliction surpassing in its evil consequences and present agony, this dreadful, spasmodic horror of water, whose very presence throws the strongest man into immediate convulsions; and which has for centuries baffled the closest investigations of the learned, and overcome the most powerful constitutions of the healthy and energetic. Ovarian dropsy is a disease not unfrequently met with in practice; and though absorption may carry off some of this extra, watery fluid, and other remedies temporarily free the sufferer, water still accumulates, and at length assumes an unchecked sway. Gradually it pervades the entire system, crawls up the intestinal canal, disturbs the respiration, deranges the stomach, and death follows as victor.

How exquisite is the provision of nature! How indicative of a Higher Power is the "Bag of Waters," in which the infant yet unborn is carried, protected from the sudden blow of accident or outward violence. How utterly indispensable for fœtal life; how impossible for parent or child otherwise to go on till the proper time of parturition.

As a proof of the importance of preserving the waters as long as may be, during the first stage of labor, I quote the following:

"Si la texture des membranes est lâche, leur rupture dès le commencement du travail. Chez quelques femmes, les eaux s'écoulent avant qéulles aient été averties par des douleurs, des effort contractiles de la matrice. Dans quelques cas, on a vu le travail tarder huit á neuf jeurs â se déclarer."*

When that all important period has arrived, and nature calls for a commencement on the part of some one, how responsive to this appeal and how gentle is the persuasive, wedge-like pressure brought to bear upon the os, dilating the cervix by its gravity, and at the same time irritating the nerves distributed to that part; thereby summoning the uterus to contract with all powerful efforts, and give forth what had lain concealed for nine long months of gradual, placental life. All who have attempted version in mal-presentation, or when the exhaustion of the parturient female calls for immediate delivery, can testify to the incalculable benefit and incomparable assistance to be derived from the "Bag of Waters." By this means, with great rapidity and moderate facility, the child may be turned, the uterus being kept back for the time by the distended sack filled with the miconium. But how impossible and vain are the attempts of the ablest

---

* **Dictionaire des sciences médicales.**

accoucheur, who, attended by no unnatural obstruction, endeavors to manipulate, when the arm becomes paralysed by the almost incredibly powerful contractions of the womb; so that at times it becomes the duty of the assistant to deliver first the doctor, and then the fœtus. The empyric can not fully appreciate the wonderful assistance of this bag, but the man of experience preserves it till the last moment, and well might the child born with a "caul" be esteemed most fortunate.—*Medical and Surgical Reporter.*

